# Supplementary material for: Forecasting influenza in Europe using a metapopulation model incorporating cross-border commuting and air travel
Source: PLoS Comput Biol. 2020 Oct 14;16(10):e1008233. doi: 10.1371/journal.pcbi.1008233 (PMC7588111; doi:10.1371/journal.pcbi.1008233)
Supplement: S1 Text — (PDF) [file pcbi.1008233.s001.pdf]

S1 Text. Supplementary methods and results.

SUPPLEMENTARY METHODS

Influenza Data Processing

The syndromic data used for this study were ILI data except in Germany and Luxembourg, which preferentially reported ARI data, as well as France, which reported ARI data during the 2012-13 and 2013-14 seasons. No virologic (FluNet) data were available for France for the 2010-11 and 2011-12 seasons. Additionally, data from Czechia in 2013-14 and Poland in 2011-12 were removed from consideration because the attack rates of total syndromic+ (including all (sub)types) for these outbreaks were less than 5% of the attack rate of the largest outbreak in these countries, an exclusion criteria laid out in [1].

Travel Data Processing

Air Data: Because air travel data are collected from both source and destination countries, the number of passengers traveling each route is, theoretically, reported twice. Due to close agreement between the data reported by source and destination countries, we chose to simply use the data as reported by source countries. Data were averaged over all years in order to preserve data along routes where data were available some years but not others. Because travel was assumed to be symmetric, if data along a route were missing in one direction but not the other, the available value was assumed to be the travel rate in both directions.

Commuting Data: The European Union (EU) Labour Force Survey is a survey of private households in 35 countries, including all EU member states. Data collection is the responsibility of individual countries, so while questions and definitions remain constant across all countries, sampling strategies may not, and results are therefore not perfectly comparable between countries [2,3]. Of the routes where no data were reported, all but four were unreported due to being below threshold 'a' (see main text) and were not simply missing. We note that these routes tended to be between countries that were geographically distant, or else represented routes where economic incentive to

commute was low [4,5]. Finally, although Ireland had reliable incoming and outgoing commuting data, the United Kingdom did not. Removing the United Kingdom from the model left Ireland isolated geographically, and we therefore subsequently removed Ireland from consideration as well.

Type and Subtype Dynamics in Europe

For each season and country, we calculated the percentage of positive influenza tests in the FluID data that were H1N1, H3N2, un(sub)typed A, and B. After allocating un(sub)typed influenza A proportionally to H1N1 or H3N2, the resulting (sub)type-specific syndromic+ data for each country over the study period can be seen in S1 Fig. Although the proportional contribution of each (sub)type by season is broadly similar across countries, substantial differences do occur. For example, during the 2014-15 season, A(H1) was the dominant (sub)type in Italy (although A(H3) was also present), while A(H3) dominated in most other countries. In the 2015-16 season, A(H1) and B circulated in all countries, but the relative activity of the two (sub)types varied by country.

Network Model

The full equations for the network model during daytime are:

$$\frac{dS_n^k}{dt} = \frac{N_n^k - S_n^k - I_n^k}{L} - \frac{\beta_n(t) S_n^k I_n}{N_n} - \frac{S_n^k}{N_n} \sum_{m \neq n} air_{Adj} r(n,m) + \frac{N_n^k}{N_n} \sum_{m \neq n} \left[air_{Adj} r(m,n) \sum_h \frac{S_m^h}{N_m} \right]$$

$$\frac{dI_n^k}{dt} = \frac{\beta_n(t) S_n^k I_n}{N_n} - \frac{I_n^k}{D} - \frac{I_n^k}{N_n} \sum_{m \neq n} air_{Adj} r(n,m) + \frac{N_n^k}{N_n} \sum_{m \neq n} \left[air_{Adj} r(m,n) \sum_h \frac{I_m^h}{N_m} \right]$$

where any model state Y_n^k is the number of susceptible or infected people who live in country *k* and work in country *n*, Y_n is the number of susceptible or infected people currently in location *n*, β_n is the transmission rate in country *n*, and $r(n,m)$ is the daily rate of air travel from country *n* to country *m*; all other parameters are as described in the main text. During nighttime, the equations are:

$$\frac{dS_n^k}{dt} = \frac{N_n^k - S_n^k - I_n^k}{L} - \frac{\beta_k(t) S_n^k I^k}{N^k} - \frac{S_n^k}{N^k} \sum_{h \neq k} air_{Adj} r(k,h) + \frac{N_n^k}{N^k} \sum_{h \neq k} \left[air_{Adj} r(h,k) \sum_m \frac{S_m^h}{N^h} \right]$$

$$\frac{dI_n^k}{dt} = \frac{\beta_k(t) S_n^k I^k}{N^k} - \frac{I_n^k}{D} - \frac{I_n^k}{N^k} \sum_{h \neq k} air_{Adj} r(k,h) + \frac{N_n^k}{N^k} \sum_{h \neq k} \left[air_{Adj} r(h,k) \sum_m \frac{I_m^h}{N^h} \right]$$

where Y^k is the number of susceptible or infected people currently in location *k*, and the other states and parameters are as described above. Because individuals are assumed to spend 8 hours in the country where they work, and 16 in their home country, daytime equations are multiplied by 1/3, and nighttime equations by 2/3.

Scaling Factors

The use of scaling factors in influenza forecasting is rooted in Bayes' Rule, such that:

$$p(i) = \frac{p(m)}{p(m|i)} p(i|m) = \gamma \cdot (syndromic+)$$

where *p(i)* is the probability of influenza infection (the quantity estimated by our model), *p(m)* is the probability that one seeks healthcare for any reason, *p(m|i)* is the probability of seeking healthcare among those with an influenza infection, and *p(i|m)* is the probability that one is infected with influenza given that one sought healthcare. This final quantity is analogous to our syndromic+ plus measure, which estimates the number of influenza cases at a given time based on data collected from those seeking healthcare for ILI or ARI. The scaling factor, *γ,* therefore represents the probability of seeking medical attention for any reason, divided by the probability of seeking medical attention conditional on infection with influenza, quantities that may be expected to vary based on surveillance systems, disease severity, and health-seeking behavior [6,7]. By multiplying syndromic+ data by such scaling factors, we are theoretically calculating the probability of influenza infection, which is equivalent in form to the output of our models, and allows the appropriate use of the EAKF. However, as explained in the main text, the WHO data

are typically reported as counts, not rates. Thus, our scaling factors must also account for differences in country population sizes and the size of surveillance catchment areas, and we expect our scaling factors to vary considerably by country. Indeed, scaling factors range from 0.027 (France, A(H3), 2016-17) to 99.17 (Luxembourg, A(H3), 2016-17) (S1 Table).

France reported ARI data for the 2012-13 and 2013-14 seasons, and ILI data for all subsequent seasons. Because of the difference in magnitude of the two data types (see S1 Fig), scaling factors were calculated separately for ARI and ILI data. Because outbreaks of influenza B occurred in the 2012-13 season but not in 2013-14, use of the leave-one-out procedure for calculating scaling factors described in the main text was not possible. Instead, the scaling factor for (sub)type B in France for the 2012-13 season was simply chosen to yield an attack rate of 15% for the season.

Observational Error Variance

Use of the EAKF requires that the degree of error in both the simulated model output and in the observations are specified. While the model error can easily be calculated as the variance of the 300 ensemble members, the error in our observations is unknown. We specify the observational error variance (OEV) at time *t* as:

$$OEV_t = b + \frac{\left(\sum_{j=t-2}^{t} \frac{O_t}{3}\right)^2}{c}$$

where O_t is the scaled syndromic+ observation at time *t*. Here, the parameters *b* and *c* were set to 1e5 and 10, respectively, based on a preliminary grid search, but can be altered for different data sources [7].

SUPPLEMENTAL RESULTS

Filter Performance

Filter divergence: To determine whether substantial filter divergence occurred during the outbreak period, we plotted the median and 50%/95% credible intervals of the ratio of the OEV (see above) to the prior ensemble variance across observed lead weeks -8

through 8 (i.e., from eight weeks prior to the observed peak through 8 week post-peak) for both the metapopulation and isolated models. Broadly, filter divergence is occurring when the OEV is substantially higher than the prior ensemble variance. For the metapopulation model, we observed that the ratio of OEV to prior ensemble variance tended to be slightly higher than one early in the season, decreased to about one as the peak approached, then increased again after the peak had passed. While there was evidence of filter divergence, this typically did not occur until several weeks after the peak had passed and was almost never encountered before five or six weeks post-peak (S2 Fig A). In other words, filter divergence was rarely an issue during the time period considered throughout this work (up to four weeks post-peak). The ratio was consistently lower for the isolated model (S2 Fig B), indicating that substantial filter divergence is less likely to occur in this model.

Cross-ensemble correlations: As explained in the main text, the EAKF adjusts unobserved model states (*S, I,* and *newI* for each of 144 individual compartments) and parameters (R_{0max}, R_{0diff}, *D, L,* and air_{Adj}) based on the cross-ensemble covariances between these states and parameters, and the inferred incidence for the 12 countries in the model (*X*). If unobserved states and parameters are being meaningfully adjusted by the filter, we will observe a non-zero cross-ensemble correlation between these states and parameters and the observed model state variables. To assess the performance of the EAKF in this work, we calculated the cross-ensemble Pearson's correlation coefficients over time between the observed and unobserved model states and parameters in the metapopulation model. Specifically, we explored the correlations between each of the five model parameters (R_{0max}, R_{0diff}, *D, L,* and air_{Adj}) and country-level incidence for all 12 countries. We also explored the cross-ensemble correlations between country-level incidence for each country, and values of *S, I*, and *newI* in each country's largest compartment (i.e., the compartment consisting of those who both work and live in that country).

The median correlation coefficients, as well as 50% and 95% credible intervals, across all (sub)types and countries throughout the influenza season can be seen in S3 Fig. Correlations between the observed state variables and both *I* (S3 Fig B) and *newI*

(S3 Fig C) were particularly high, which was expected given that the modeled country-level incidence is highly dependent on *newI* among those who live and work in a given country. Correlations with *S* (S3 Fig A) and *D* (S3 Fig E) were also consistently high, and small positive associations between the observed state variables and R_{0max} (S3 Fig F) were observed, particularly early in the season, indicating that the filter worked to adjust values for these states and parameters over time. Correlations were small, on the other hand, for R_{0diff} (S3 Fig G), and remained around zero for the duration of the season for both *L* (S3 Fig D) and air_{Adj} (S3 Fig H), indicating that the filter did little to adjust these values. This, however, is to be expected: *L* represents loss of immunity on time scales of years, and its influence is difficult to observe over the course of a single influenza season, while air_{Adj} merely allows for small differences in the magnitude of air travel. Overall, our findings confirm that the EAKF effectively works to adjust unobserved states and parameters in our metapopulation model, and agree broadly with our synthetic test results (see below). Results were similar for the isolated model and are not shown.

Descriptive Statistics by (Sub)type

The number of countries where outbreaks occurred during a (sub)type-specific seasonal influenza outbreak ranged from 8 to 12 countries (mean = 10.8 median = 11.5) for A(H1), from 8 to 12 countries (mean = 9.6, median = 10.0) for A(H3), and from 2 to 12 countries (mean = 9.3, median = 11.5) for B. Therefore, it appears that the number of countries experiencing outbreaks did not differ notably by subtype, with the exception of a season where at least 4 countries reported over 10% positivity rates for influenza B, but only two countries experienced any outbreak onset as determined using scaled syndromic+ observations. We note that this outbreak of B influenza was particularly late in the season (onsets at weeks 56 and 58), and that the number of clinical cases was already quite low by this point in the outbreak, which explains the lack of onsets despite positivity rates being above our threshold of 10%.

Within an outbreak, the time between the earliest and latest outbreak onsets ranged from 5 to 11 weeks (mean = 7.3, median = 6.5) for A(H1), 5 to 10 weeks (mean = 8, median = 8) for A(H3), and 2 to 12 weeks (mean = 7.7, median = 7) for B; the time

between the earliest and latest outbreak peaks was between 6 and 13 weeks (mean = 8.3, median = 7) for A(H1), 5 and 11 weeks (mean = 8, median = 8) for A(H3), and 2 and 12 weeks (mean = 7.3, median = 8) for B. This suggests that, by this rough metric, outbreak synchrony between countries did not vary substantially by subtype. Finally, we found that peak timing (Kruskal-Wallis test, $p < 0.005$), but not onset timing ($p > 0.1$), differed significantly by subtype over all available seasons. More specifically, post-hoc Nemenyi tests indicate that peak timing tended to be later for outbreaks of influenza B ($p < 0.01$ for comparisons against both A(H1) and A(H3)), although we note that the median peak timing for outbreaks of influenza B in our dataset was only 1-2 weeks later than the medians for outbreaks of A(H1) or A(H3).

Because raw data are not reported as rates, it is difficult to compare peak intensity by subtype. In order to explore whether certain subtypes consistently yielded larger or smaller outbreaks, we first adjusted observed peak intensity values for each country by dividing by the peak intensity of the largest outbreak observed for that country over all seasons and subtypes. We could then assess whether these relative outbreaks sizes differed by subtype. We found that outbreaks of A(H3) influenza tended to be larger than those of A(H1) (post-hoc Nemenyi test, $p < 0.01$) or B ($p < 0.05$). This result is in agreement with previous reports that outbreaks of A(H3) tend to be larger and of higher severity than outbreaks of A(H1) [8].

Additional Model Fit Results

As described in the main text, model fits for A(H3) and B influenza during the 2012-13 season are visualized in S4 Fig.

Results by Observed Lead Week

Generally, forecasting results were similar whether they were assessed by predicted (Fig 3) or observed (S5 Fig A-C) lead week. This remained true if season-country-subtype-week pairs were removed because either the network or the isolated models did not predict an onset (S5 Fig D-F). Interestingly, if we only include pairs for which both the network and isolated models produce forecasts predicting an onset, the observed improvement in onset timing accuracy forecasts appears to shrink. This

suggests that any improvement the network model offers for onset timing predictions is not due to the production of more skillful forecasts, but rather due to the recognition of upcoming outbreak onsets not picked up by the isolated model.

To ensure that our decision to remove forecast pairs where either the network or the isolated model did not predict an onset was not overly aggressive, we repeated our analysis, this time including all forecasts for outbreaks with an observed onset. In other words, forecasts where no onset was predicted were not removed. We found that the isolated forecasts performed significantly better for both peak timing and peak intensity ($p < 0.01$), but not for onset timing ($p > 0.15$). Thus, results were consistent with analyses in the main text for onset timing, but not for peak timing or peak intensity. However, we note that while the main text results were not statistically significant, they still displayed a tendency to favor the isolated model: the isolated model outperformed the network model for a greater number of country-season-(sub)type-week pairs for 999 of the 1000 random combinations of runs for peak timing, and for 940 of the 1000 random combinations for peak intensity. Furthermore, we emphasize that the magnitude of the difference in the mean log scores between the network and isolated models remained minimal, especially for peak intensity (S5 Fig G-I); thus, these differences in significance are likely due at least in part to the increase in sample size.

Results by (Sub)type

Results by (sub)type are described in the main text under “Retrospective Forecast Accuracy.” Plots of log scores by predicted lead week separated by (sub)type are found in S6 Fig.

Results by Country

Log scores for peak timing and intensity by country by predicted lead week can be seen in S7 Fig. Because so few forecasts of onset timing were produced prior to outbreak onset, we refrain from plotting these results. Although the network model offered no improvement in forecast accuracy overall, we wondered whether improvement may be observed for specific countries. In particular, we might expect that countries with lower-quality data may benefit from the inclusion of other countries with

higher-quality data, or that countries with larger commuting flows may benefit more from a network model like the one developed here. However, there is generally very little difference between network and isolated model results in most countries, mirroring the results in the main text. Additionally, Luxembourg, the country with the least-smooth data (see “Data Quality by Country” below), is one of the countries for which the network model clearly degraded peak timing forecast accuracy (the others being Hungary, Italy, and Poland). Czechia and the Netherlands appear to see the most improvement in forecast accuracy when the network model was used, but these improvements are small. Overall, no clear patterns emerged concerning network and isolated model performance by country.

Results (Mean Absolute Errors)

In addition to log scores, we assessed forecast results using mean absolute errors (MAE), a metric which accounts for accuracy but not for certainty or calibration. MAE is calculated by taking the absolute value of the difference between the predicted and observed values of a given metric for all forecasts, then calculating the mean value over all forecasts. Note that, because values for peak intensity varied greatly between countries, we instead calculated mean absolute percentage errors (MAPE) for peak intensity; here we divide the absolute difference by the observed peak intensity value before taking the mean.

When assessed by predicted lead week, the network model appears to improve forecasts of both peak and onset timing prior to the predicted peak or onset, although we again note that the number of forecasts generated prior to outbreak onsets was small (S8 Fig A and C). Peak intensity, however, appears to be more accurately forecasted by the isolated models, particularly at lead weeks -4 to -3 (S8 Fig B). Thus, results for onset timing are consistent with those found in the main text using log scores (Fig 3C). Peak timing appears to be more accurately predicted by the network model prior to the predicted peak when assessed using MAE, yet received lower log scores than the isolated model over the same range of lead weeks (Fig 3A; although we note that the difference in log scores was not statistically significant). This suggests that network forecasts of peak timing may be closer to the observed values on average, but

place less confidence in their forecasts (i.e., fewer ensemble members fall into the observed bin). Meanwhile, network model forecasts of peak intensity were noticeably less accurate than isolated model forecasts several weeks before the peak when assessed using MAPE, but the two models had similar log scores at these lead weeks (Fig 3B). Results using MAPE are, however, consistent with our sensitivity analysis above, which found that, when all forecasts are included, log scores are significantly lower for the network model forecasts (S5 Fig H).

MAEs and mean absolute percentage errors (MAPE) separated by (sub)type can be found in S9 Fig. Network model improvements for peak timing forecasts appear greatest for influenza B (S9 Fig C), while improvements for onset timing are primarily found for (sub)type A(H1) (S9 Fig G). As in S8 Fig, the network model appears to degrade peak intensity forecasts, with the potential exception of very early forecasts of A(H3) (S9 Fig E). However, as with the results for all (sub)types combined, the magnitude of these differences is quite small.

Alternative Calibration Plots

If forecasts are properly calibrated, we expect the distribution of forecast errors to have a mean of 0; otherwise, the forecasts are biased. S10 Fig compares the distribution of forecast errors for peak timing and intensity for the network and isolated models. Note again that relative errors were used for peak intensity, to control for differences in intensity by country. We see little difference between the models in terms of bias. Both models appear to produce relatively unbiased estimates of peak timing at all lead week ranges, with the distributions becoming narrower as the peak approaches and passes. Estimates of peak intensity, on the other hand, appear biased low at early lead weeks, particularly for the isolated forecasts, but appear relatively unbiased starting at predicted lead week -4, and become more precise at later leads. Overall, these results agree with the finding in the main text that both the network and isolated models are well-calibrated for forecasting peak timing and intensity (Fig 4).

SUPPLEMENTARY ANALYSES

Synthetic Testing

In order to test whether our model is capable of fitting realistic influenza outbreaks, we conducted synthetic testing, wherein we test the model-inference system's ability to properly fit model-generated "outbreak observations." This is done because, unlike for observed outbreaks, we know the exact parameter and initial state values that were used to generate synthetic observations, allowing assessment of the accuracy of the model fitting.

We drew 1000 random combinations of initial state and parameter values from realistic ranges (see main text) using Latin Hypercube Sampling. Unlike for forecasting, initial values of *S* and *I* were chosen for each country rather than for each individual compartment. *S0* in each commuting compartment was then chosen from a normal distribution around *S0* for those living and working in the commuters' home country with standard deviation 0.025. Initial infected numbers in a country were distributed among all compartments sharing a home country according to relative population size. These state and parameter combinations were then run forward for the duration of an influenza season (52 weeks). Commuting flows were set to be the mean number of commuters across all seasons; missing routes were filled as described in the main text. Realistic outbreaks were determined to be those where: a) at least 7 of 12 countries had outbreak onset (defined as in the main text), and b) no more than 1 of those countries with onsets had peak timings before week 52 or after week 12. These metrics were based on preliminary exploration of the syndromic+ observations for each (sub)type. We selected five outbreaks on which to perform synthetic testing. As influenza outbreaks in Europe tend to move from west to east, we chose four outbreaks that progressed from west to east (one strongly and three weakly), and one that moved east to west instead (as observed during the 2015-16 season). Parameter values for these outbreaks can be found in S4 Table.

Because observed data are rarely as smooth as synthetically-generated data, we added random error to our synthetic observations. Specifically, we added normally-distributed error to each point with a mean of zero and standard deviation equal to the square root of the observational error variance at that point, calculated as described

above under “Ensemble Adjustment Kalman Filter,” with b = 1e5 and c = 10. Any resulting negative values were set to zero.

We then fit our network model to each of the 5 error-laden synthetic outbreaks, and compared the inferred and true values of the model parameters D, R_{0max}, and R_{0diff}; and the composite model parameters β, R_0 (as defined in Equation 2 in the main text), and R_{eff} (the effective reproductive number, or the average number of secondary cases caused by a single initial case, taking population susceptibility into account; calculated as $R_0(\frac{S}{N})$). Because R_{eff} dictates the course of the epidemic, it is particularly important that the model be able to correctly infer its value. Comparisons were achieved by calculating errors relative to the true values at all time points.

Fits for D, R_{0max}, and R_{0diff} over the course of the five synthetic outbreaks can be found in S11 Fig. We found that the model-inference system fit D relatively well in most cases, with a slight tendency to underestimate its value. R_{0max} and R_{0diff} appear more difficult to accurately infer, with the model settling on similar inferred values for all five synthetic outbreaks. This may suggest that the model is not particularly sensitive to values of these two parameters, or that multiple combinations of R_{0max} and R_{0diff} are capable of producing the same outbreak patterns.

The distribution of relative errors for β, R_0, and R_{eff} for all countries and synthetic outbreaks at timepoints 5, 10, 15, and 20 are shown in S12 Fig. Generally, the inferred values of β, R_0, and R_{eff} approached the true values over time. R_0 was fit quite closely at all timepoints (S12 Fig B), while the model seems less capable of correctly inferring β (S12 Fig A). Encouragingly, although R_{eff} tended to be underestimated early in the season, it was well-fit during the outbreak itself (S12 Fig C). That said, the tendency of the model to underestimate R_{eff} before outbreak onset could be why so few early forecasts of onset timing were produced (Table 1).

Data Quality by Country

While small differences in data quality are not necessarily associated with forecast accuracy [1,7], we nonetheless expect that particularly poor-quality data will be more difficult to fit, and will produce lower-quality forecasts. We briefly assessed data

quality by country and subtype, and its association with fit and forecast accuracy using two metrics:

1. The proportion of weeks within an outbreak where no data are available (where an outbreak is defined as starting in week 40 and ending in week 19 of the following year)
2. Lag-one autocorrelation by outbreak (a measure of signal smoothness)

For these analyses, only countries with an observed onset were included for each (sub)type and season.

First, we note that the two metrics are not significantly correlated with one another, and in fact trend towards being slightly negatively associated (Kendall's tau = -0.101, $p < 0.07$), emphasizing that there is no one measure that perfectly encapsulates data quality. Missingness (metric 1) ranges from 0 to 37.5% of time points during a season (mean = 9.67%, median = 6.25%), whereas smoothness (metric 2) ranges from 0.29 to 0.95 (mean = 0.83, median = 0.87), suggesting high variability in data quality, and confirming that both missingness and noisiness are prevalent in our data.

Both metrics above differ significantly by country (Kruskal-Wallis test, $p < 0.0001$ for both metrics). Briefly, Germany has particularly low missingness, and Italy has high missingness; Luxembourg has notably low signal smoothness. Because all subtypes make use of the same clinical and virologic data points, it is not meaningful to compare missingness by subtype. Signal smoothness does not differ significantly by subtype (Kruskal-Wallis test, $p = 0.45$).

Finally, we assessed whether either of these metrics were associated with model fit (measured by RMSE) or forecast accuracy (measured by log score). First, RMSE values were averaged over all runs for each country-season-subtype combination, and log scores were averaged over all runs and lead weeks (predicted leads -6 through 4). Because we are particularly interested in forecast accuracy prior to the peak, we also looked at log scores averaged over predicted leads -6 through -1 only. Neither RMSEs nor log scores were significantly associated with missingness. Smoothness was positively associated with log scores for peak timing accuracy before the predicted peak, but only for the isolated models (Kendall's tau = 0.124, $p < 0.02$); no other significant associations between smoothness and either RMSEs or log scores were found.

Therefore, at least using these simple metrics of data quality, we found little evidence that data quality differences within our dataset were associated with network model fit quality and forecast accuracy.

Binary Onset Forecasts

While this work is primarily concerned with the quality of forecasts for specific outbreak timepoints, given that an outbreak does indeed occur, it is worth examining whether or not our forecasting system is capable of correctly predicting whether or not an outbreak will occur at all. Because outbreaks for a season-(sub)type pair rarely occur in all countries, an ability to predict any outbreak is relevant throughout the season. For example, if we observe outbreaks in six of the twelve countries, we may still wonder which of the remaining 6 countries will go on to have an outbreak, and which will not.

We examined the sensitivity and specificity of predictions of any versus no onset by calendar week. Because the model forms used here are likely to produce unreliable forecasts in the absence of some influenza signal, we removed forecasts generated before the week of the first observed onset for each season and (sub)type.

We found that sensitivity was low at earlier calendar weeks but increased over time (S13 Fig A), indicating that the models become better at correctly identifying outbreaks with an observed onset at later weeks. This increase in sensitivity, however, was primarily due to an increase in models correctly identifying observed onsets that have already occurred; when we limited the analysis to forecasts produced on or before the week of an observed onset, sensitivity remained low (mean = 17.0% and median 12.7% across both models and all lead weeks) even at later lead weeks. This is unsurprising given our results in the main text, which indicated that both the network and isolated models rarely predict the occurrence of an onset prior to the onset itself (see Table 1). Specificity, on the other hand, was uniformly high (S13 Fig B), meaning that forecasts predicting an outbreak were rarely generated for seasons, (sub)types, and countries where no outbreak ultimately occurred. Altogether, these findings suggest that forecasts of an upcoming outbreak are unlikely to be false positives, but that forecasts predicting no outbreak onset have little predictive power. We also note that, as in the

main text results, we found little difference between results from the network and isolated models.

Inferred States and Parameters

If a model can only produce appropriate estimates and forecasts by inferring unrealistic values of model states (the number susceptible, infected, and newly infected) and parameters (here, R_{0max}, R_{0diff}, L, D, and air_{Adj}), it suggests that the model itself may not be well-specified. We therefore assessed whether the values of unobserved model states and parameters inferred by the network model were realistic. Additionally, we explored state and parameter patterns by (sub)type. In addition to the model parameters listed, we also looked at inferred values of R_0 (the basic reproductive number), and R_{eff}.

The inferred value of $S0$ for a given country during a (sub)type-specific outbreak was taken to be the maximum inferred proportion susceptible over the time period beginning with the first onset observed in that (sub)type-specific outbreak, and ending in week 19 (i.e., the end of the season). Weeks prior to the first onset were removed because, in synthetic testing (see above), the proportion susceptible was often overestimated during the first few weeks of fitting, particularly in countries with low $S0$. The maximum R_{eff} for a country during a given (sub)type-specific outbreak was found using the same method. R_0 was taken as the value of R_0 during the week with maximum R_{eff}. Because our model assumes a single pathogen, we expect R_0 to vary between countries only according to differences in absolute humidity. Thus, after confirming that no significant differences existed for R_0 by country (Kruskal-Wallis test, as described in next paragraph; all $p > 0.05$), we calculated an overall estimate of R_0 for each (sub)type-specific season by taking the mean R_0 over all countries. The five model parameters were simply assessed at week 7, twenty weeks after fitting begins.

Each season and (sub)type was fit five times, with each run having different initial conditions and commuting matrices (see main text). Because these runs were not independent, we randomly chose a single run for each country-season-(sub)type combination (season-(sub)type combinations for R_0 and model parameters) before checking for significant differences using Kruskal-Wallis rank sum tests. This process

was repeated 100 times, and differences were considered significant if at least 50 of the random permutations yielded p-values less than 0.05 (see S1 Text from [1]).

Estimates for *S0* ranged from 55.6% to 94.4% of a country's population (58.1% to 94.4% among those countries, seasons, and (sub)types where an onset was observed), and maximum R_{eff} fits ranged from 0.68 to 2.21 (0.94 to 2.21 where onsets occurred). We found that estimates of maximum R_{eff} differed significantly by (sub)type (S14 Fig B). Post-hoc Nemenyi tests using a Bonferroni correction for multiple comparisons, also performed on 100 permutations of the estimates, revealed that inferred maximum R_{eff} values trended higher for A(H3) than for A(H1), results that are in line with our finding above that A(H3) outbreaks in our dataset tended to be larger than those of other (sub)types (see Descriptive Statistics by (Sub)type). While the initial Kruskal-Wallis test suggested a significant difference in *S0* by country, post-hoc Nemenyi tests found that none of the pairwise comparisons were significant once a Bonferroni correction was applied. That said, Kendall's rank correlation suggested that countries that are further east and further north (using the latitude and longitude of each country's capital city) tended to have lower *S0*; this pattern may reflect the model's attempt to fit outbreaks that tend to move from west to east. No significant differences in *S0* were observed by (sub)type (S14 Fig A).

Generally, parameters common to all countries also fell within realistic ranges [9–13], with values ranging from 1.22 to 2.42 for R_0, from 2.56 to 10.39 days for *D*, and from 4.17 to 10.58 years for *L*. Estimated values for air_{Adj} fell between 0.79 and 1.31, although we note that neither air_{Adj} nor *L* were inferred particularly well in synthetic testing, and results for these parameters should be interpreted with caution. Using Kruskal-Wallis tests as described above, none of these parameters were significantly associated with (sub)type. However, we note that, since only a single value of each parameter is estimated for each season-(sub)type combination, the "sample size" here is quite small. In particular, there seems to be a trend toward higher estimates for *D* during outbreaks of A(H3) and B as compared to A(H1) (S14 Fig D).

REFERENCES

[1] Kramer SC, Shaman J. Development and validation of influenza forecasting for 64 temperate and tropical countries. PLOS Comput Biol 2019;15:e1006742. https://doi.org/10.1371/journal.pcbi.1006742.

[2] EU labour force survey - main features and legal basis. Eurostat n.d. https://ec.europa.eu/eurostat/statistics-explained/index.php?title=EU_labour_force_survey_%E2%80%93_main_features_and_legal_basis.

[3] European Commission. The European Union labour force survey - Methods and definitions - 2001 2003.

[4] Mathä T, Wintr L. Commuting flows across bordering regions: a note. Appl Econ Lett 2009;16:735–8. https://doi.org/10.1080/13504850701221857.

[5] Decoville A, Durand F, Sohn C, Walther O. Comparing Cross-border Metropolitan Integration in Europe: Towards a Functional Typology. J Borderl Stud 2013;28:221–37. https://doi.org/10.1080/08865655.2013.854654.

[6] Shaman J, Karspeck A, Yang W, Tamerius J, Lipsitch M. Real-time influenza forecasts during the 2012–2013 season. Nat Commun 2013;4. https://doi.org/10.1038/ncomms3837.

[7] Morita H, Kramer S, Heaney A, Gil H, Shaman J. Influenza forecast optimization when using different surveillance data types and geographic scale. Influenza Other Respir Viruses 2018. https://doi.org/10.1111/irv.12594.

[8] Park J-E, Ryu Y. Transmissibility and severity of influenza virus by subtype. Infect Genet Evol 2018;65:288–92. https://doi.org/10.1016/j.meegid.2018.08.007.

[9] Carrat F, Flahault A. Influenza vaccine: The challenge of antigenic drift. Vaccine 2007;25:6852–62. https://doi.org/10.1016/j.vaccine.2007.07.027.

[10] Chowell G, Miller MA, Viboud C. Seasonal influenza in the United States, France, and Australia: transmission and prospects for control. Epidemiol Infect 2008;136. https://doi.org/10.1017/S0950268807009144.

[11] Truscott J, Fraser C, Hinsley W, Cauchemez S, Donnelly C, Ghani A, et al. Quantifying the transmissibility of human influenza and its seasonal variation in

temperate regions. PLoS Curr 2009;1:RRN1125. https://doi.org/10.1371/currents.RRN1125.
[12] White LF, Pagano M. Transmissibility of the influenza virus in the 1918 pandemic. PloS One 2008;3:e1498. https://doi.org/10.1371/journal.pone.0001498.
[13] Mills CE, Robins JM, Lipsitch M. Transmissibility of 1918 pandemic influenza. Nature 2004;432:904–6. https://doi.org/10.1038/nature03063.
