# Supplementary material for: Forecasting influenza in Europe using a metapopulation model incorporating cross-border commuting and air travel
Source: PLoS Comput Biol. 2020 Oct 14;16(10):e1008233. doi: 10.1371/journal.pcbi.1008233 (PMC7588111; doi:10.1371/journal.pcbi.1008233)
Supplement: S1 Table — Note that France switched from preferentially collecting ARI to ILI data prior to the 2014–15 season; scaling factors for France are listed as ARI/ILI. (PDF) [file pcbi.1008233.s020.pdf]

S1 Table. Scaling factors by country, (sub)type, and season. Seasons/(sub)types with no outbreaks are left blank; "NA" indicates a season with missing data. Note that France switched from preferentially reporting ARI to ILI data prior to the 2014-15 season, and that scaling factors were calculated separately for seasons with ARI and ILI data.

**Country**	**(Sub)type**	2010-11	2011-12	2012-13	2013-14	2014-15	2015-16	2016-17	2017-18
Austria	A(H1)	26.30		17.53	17.53	17.53	17.53		17.53
	A(H3)		25.09	25.09	25.09	25.09		30.63	
	B	23.30		23.30		23.30	23.30	20.64	34.95
Belgium	A(H1)	12.98		12.98	12.98	12.98	12.98		12.98
	A(H3)		15.81	15.81	15.81	23.54		15.81	
	B	15.92		18.79		15.92	15.92	15.92	15.92
Czechia	A(H1)	1.22		1.22	NA	1.06	1.22		1.22
	A(H3)		0.99	1.32	NA	1.55		1.32	
	B	1.33		1.33		1.33	1.33	1.33	2.00
France	A(H1)	NA		2.08	1.95	0.074	0.069		0.074
	A(H3)		NA	3.92	3.37	0.033		0.027	
	B	NA		1.22		0.12	0.18	0.11	0.12
Germany	A(H1)	0.32		0.32	0.32	0.32	0.32		0.32
	A(H3)		0.41	0.41	0.24	0.41		0.41	
	B	0.50		0.50		0.50	0.50	0.34	0.68
Hungary	A(H1)	1.81		1.81	1.81	1.81	1.81		1.81
	A(H3)		1.18	1.18	1.18	1.34		1.18	
	B	1.19		1.19		1.19	1.19	1.19	1.79
Italy	A(H1)	2.04		2.04	2.04	2.04	2.04		2.75
	A(H3)		1.75	1.60	1.75	1.75		1.76	
	B	1.19		1.19		1.19	1.19	1.19	1.79
Luxembourg	A(H1)	83.24		83.24	58.79	83.24	83.24		83.24
	A(H3)		74.33	54.67	74.33	74.33		99.17	
	B	82.04		82.04		82.04	91.06	47.45	82.04
Netherlands	A(H1)	32.32		32.32	32.32	32.32	32.32		32.32
	A(H3)		46.89	46.89	46.89	47.93		46.89	
	B	42.13		42.13		42.13	42.13	42.13	63.19
Poland	A(H1)	2.56		2.56	2.56	2.56	3.21		2.56
	A(H3)		NA	1.68	1.68	1.68		2.51	
	B	1.65		1.65		1.65	1.65	1.65	2.48
Slovakia	A(H1)	0.58		0.58	0.58	0.58	0.58		0.58
	A(H3)		1.17	1.17	1.17	1.17		1.75	
	B	0.81		0.81		0.81	0.81	0.81	1.22
Spain	A(H1)	3.27		3.27	3.27	3.27	3.27		3.27
	A(H3)		6.35	5.16	5.16	5.16		5.16	
	B	6.86		6.86		6.86	6.86	6.30	7.38
