# Supplementary material for: Forecasting influenza in Europe using a metapopulation model incorporating cross-border commuting and air travel
Source: PLoS Comput Biol. 2020 Oct 14;16(10):e1008233. doi: 10.1371/journal.pcbi.1008233 (PMC7588111; doi:10.1371/journal.pcbi.1008233)
Supplement: S2 Table — Pairs are removed when either the network or isolated model produces no forecasts with any predicted onset. Final numbers reflect the number of forecasts included in the Friedman test analyses from the main text, and in S3D–S3F Fig. (PDF) [file pcbi.1008233.s021.pdf]

S2 Table. Number of country-season-(sub)type-week forecast pairs removed from consideration prior to conducting Friedman tests, by observed peak and onset lead week. Pairs are removed when either the network or isolated model produces no forecasts with any predicted onset. Final numbers reflect the number of forecasts included in the Friedman test analyses discussed in the main text, and in S5 Fig D-F.

	Lead Week:	-6	-5	-4	-3	-2	-1	0	1	2	3	4
Peak Lead Week	Original	164	164	166	167	167	163	169	167	164	167	167
	No predicted onset	125	106	83	61	43	25	7	7	7	9	9
	Remaining	39	58	83	106	124	138	162	160	157	158	158
	**% Remaining**	**23.78%**	**35.37%**	**50.00%**	**63.47%**	**74.25%**	**84.66%**	**95.86%**	**95.81%**	**95.73%**	**94.61%**	**94.61%**
Onset Lead Week	Original	148	156	162	159	165	152	169	169	169	166	169
	No predicted onset	147	151	160	145	150	148	99	25	11	10	11
	Remaining	1	5	2	14	15	4	70	144	158	156	158
	**% Remaining**	**0.68%**	**3.21%**	**1.23%**	**8.81%**	**9.09%**	**2.63%**	**41.42%**	**85.21%**	**93.49%**	**93.98%**	**93.49%**
