# Supplementary material for: Forecasting influenza in Europe using a metapopulation model incorporating cross-border commuting and air travel
Source: PLoS Comput Biol. 2020 Oct 14;16(10):e1008233. doi: 10.1371/journal.pcbi.1008233 (PMC7588111; doi:10.1371/journal.pcbi.1008233)
Supplement: S3 Table — (PDF) [file pcbi.1008233.s022.pdf]

S3 Table. Number of forecasts predicting any onset by predicted onset lead week, separated by (sub)type.

	**Lead Week**	**-6**	**-5**	**-4**	**-3**	**-2**	**-1**	**0**
A(H1)	Network	1	1	2	13	15	30	242
	Isolated	6	22	11	25	16	14	214
A(H3)	Network	1	13	8	10	25	39	227
	Isolated	3	11	4	12	18	9	190
B	Network	0	2	1	9	17	60	236
	Isolated	2	10	5	10	10	25	222
