# Supplementary material for: Forecasting influenza in Europe using a metapopulation model incorporating cross-border commuting and air travel
Source: PLoS Comput Biol. 2020 Oct 14;16(10):e1008233. doi: 10.1371/journal.pcbi.1008233 (PMC7588111; doi:10.1371/journal.pcbi.1008233)
Supplement: S4 Table — (PDF) [file pcbi.1008233.s023.pdf]

S4 Table. Parameters values used to generate synthetic outbreaks, and the number of countries with outbreak onsets.

Synthetic Outbreak	*L* (years)	*D* (days)	R_{0max}	R_{0diff}	*airScale*	# of Countries w/ Onsets
1	4.61	5.58	2.15	0.48	1.24	10
2	3.36	6.18	2.28	0.57	0.92	11
3	4.92	6.14	2.63	0.98	1.17	12
4	8.56	3.96	2.11	0.90	0.87	7
5	6.33	4.71	2.33	0.84	1.16	11
