# Supplementary material for: Forecasting influenza in Europe using a metapopulation model incorporating cross-border commuting and air travel
Source: PLoS Comput Biol. 2020 Oct 14;16(10):e1008233. doi: 10.1371/journal.pcbi.1008233 (PMC7588111; doi:10.1371/journal.pcbi.1008233)
Supplement: Attachment — Submitted filename: Response_to_reviewers.docx [file pcbi.1008233.s024.docx]

*Reviewer #1: This report compares ensemble predictions of influenza evolution for 12 European countries using a SIRS model that includes information about commuting and air travel and one that does not. The conclusions are that there is no apparent improvement from adding the travel information.

I have limited expertise in this type of model or application, but significant expertise in ensemble filtering and the statistical science of quantifying prediction skill and uncertainty. My comments will focus almost entirely on concerns about the clarity of the description of the filtering method and specifics of the forecast system design and validation.

My opinion is that the manuscript in its present form is not suitable for publication. There are four major issues that need addressed and it is unclear to me how difficult that will be. In particular, the filtering algorithm is not clearly described in the text and equations and fundamental filtering challenges are not mentioned or addressed. The algorithm also appears to withhold large sets of forecasts depending on a priori measures of forecast quality that are either not possible to assess in real time or depend on forecast outcome. It is nearly impossible to assess the quality of forecasts when this is done. Finally, the models appear to be tuned without cross validation using a very small set of forecast years. It is possible that all of these problems can be addressed by clarifying the text. However, I must recommend rejection for now without knowing that is the case.*

Thank you for your detailed feedback. We address each of your concerns more specifically below.

*Major comments:*

*1. The description of the EAKF is insufficiently clear and complete. Equation (3) is correct, however, X and O are not clearly defined. I assume that X includes the state variables and the parameters, but a list of the parameters that are estimated is never given and the exact size and definition of the state is not clearly state.*

We agree and have revised the manuscript to include specific descriptions of both *X* and *O* (please see lines 263-275 in the revised document), after explaining the terms in Equation 3.

*In addition, the functional relation between O and X is not clearly presented; this would be referred to as a forward operator in most EAKF applications. The section ‘choice of scaling factors’ appears to be defining the forward operators, but it is insufficiently clear. At the end, there should be equations saying X=, and O=f(X) where f is clearly defined.*

In this application, the forward operator is the metapopulation model. We have clarified this in lines 278-281.

The scaling factors, in contrast, are simply used to account for the fact that the raw influenza data are count data, while the networked SIRS model outputs information on new infections as rates. This scaling is performed prior to model fitting using the EAKF, and the resulting values are treated as rates of A/H1N1, A/H3N2, or B influenza per 100,000 population. The specific scaling values are chosen using an algorithm that we have shown to allow for skillful forecast performance for the countries modeled here in past work. We have edited the section “Choice of Scaling Factors” to help mitigate confusion (see also our responses to major comment 4 and minor comment 13).

*2. The EAKF suffers from two types of error, insufficient prior variance and overestimated correlations. These problems must be addressed by some form of localization and inflation respectively. I’m willing to give the authors some slack on localization given that they use fairly large ensembles (I think, although there is the question of whether they are rejected even in the EAKF analysis).*

You are correct that we have elected not to use any form of localization in this work. Because there is human movement between countries in the form of commuting and air travel, we feel it is appropriate that adjustments to state variables take into account observations from all other location in the model. In the case of our parameters, which are constrained to be equal in all locations, localization is not an issue.

Individual ensemble members are never themselves rejected, so all model runs and forecasting results refer to forecasts consisting of 300 ensemble members. We have clarified this in the main text (see lines 415-417).

*Still, they should make some sort of statement about the magnitude of correlations between their observations and state/parameters to give confidence that the assimilation is meaningful.*

Thank you for your suggestion. We have performed a supplementary analysis on how the observed model states and unobserved states and parameters of both the network and isolated models correlate over time (see lines 295-299, as well as “Filter Performance: Cross-ensemble correlations” in S1 Text and S3 Fig).

*Inflation is a much bigger issue here since there is clearly large model error and parameters without evolution equations are being estimated. This will certainly lead to insufficient variance and almost certainly to filter collapse/divergence for the application. The authors make only one passing mention of this, provide no evidence that it is not happening (and suggest that it is), and provide no discussion of why they are able to avoid dealing with a problem that is almost universal. I think it is unlikely that this EAKF application is statistically valid without inflation and I need some sort of evidence of consistency between prior ensembles and observations to convince me otherwise. There is a vast literature on this subject and something needs to be cited.*

Filter divergence was discussed in S1 Text, where we stated that we multiplicatively inflate prior ensemble variance by 1.05 at each time step. However, we agree that this is too important a point to be relegated to the supplementary materials, and have moved our discussion of filter divergence to the main text (lines 285-292); we now also include a citation to Anderson’s 2001 paper on the EAKF (see below). Furthermore, we demonstrate in a new supplementary analysis that the ratio of observational error variance to prior ensemble variance does not increase substantially until several weeks after the observed outbreak peak for both the network and isolated models, suggesting that substantial filter divergence does not occur during the lead weeks we consider throughout this work (see “Filter performance: Filter divergence” in S1 Text, as well as S2 Fig).

Anderson JL. An Ensemble Adjustment Kalman Filter for Data Assimilation. Mon Weather Rev. 2001 Dec 1;129(12):2884–903.

*3. The authors appear to withhold forecasts based upon criterion that either cannot be computed in real-time or that are conditional on the desired result. This generally results in residual forecast sets that cannot be evaluated with simple statistical tests. The first example occurs in the section ‘choice of seasons’. Forecasts are not made if the positivity rate is low. This can be computed in realtime, but why exclude these forecasts? If the model has predictive capability, it should respond by keeping rates low, unless they are going to get high. What can we conclude about the model if it is not able to do this correctly?*

In this work, we are primarily interested in forecasting key points in an outbreak, specifically peak timing, peak intensity, and onset timing, and not whether or not an outbreak will occur. As these key timepoints do not occur in the absence of an outbreak, we are not interested in forecasts produced for (sub)types that do not produce widespread outbreaks during a given season.

Perhaps more to your point, in this work we use a relatively simple networked, compartmental SIRS model to perform forecasts. The SIRS model has a limited suite of behaviors; specifically, it has a tendency to produce single-peak outbreaks. Thus, while it can be good tool for forecasting the specifics of a given outbreak (onset and peak timing, peak intensity), it is less appropriate for predicting whether or not an outbreak will occur at all, especially when there is little influenza signal in the observations (although please see the additional supplementary analysis described under minor point 18).

More specifically, when influenza positivity rates are low (whether because it is still early in the influenza season, or because a given (sub)type is not showing substantial activity), observations are overwhelmingly zero or near zero. Thus, the signal in the observations is insufficient to reliably inform key model parameters. While some ensemble members likely will project a flat trajectory, keeping rates low, others will likely predict outbreaks of varying sizes and durations (see the paragraph above), resulting in unreliable forecasts with wide credible intervals. Indeed, we know from past work that, when signal is low, forecasts using model-inference systems like that described in this work are outperformed by forecasts based on historical expectance. Because we are able to assess in real-time whether or not the observations exhibit sufficient signal for reliable forecasting, we choose to perform and assess forecasts for a given (sub)type and season only if the signal in the observations reaches the thresholds specified under “Choice of Seasons.”

*A more serious example occurs around line 335. Forecasts are only compared for initial conditions in a fixed interval around a specific verification criterion. It appears that this condition cannot be computed in real-time in some instances.*

You are correct that the observed lead week cannot be calculated in real-time, which we have made clearer in the main text (line 392). For this reason, figures demonstrating forecast performance (Figures 3 and 4) are plotted by predicted lead week instead, which can be calculated in real-time. However, we use observed lead week in our statistical analyses because it allows us to conduct a paired statistical analysis of the results: for each model run, a single forecast is produced for each lead week, (sub)type, season, and country for both the network and isolated models. This is not true for predicted lead week. Thus, using observed lead week allows for a more rigorous statistical analysis.

The time window of six weeks prior to the peak through four weeks after allows for model assessment before and after the peak at lead times that may be useful to public health practitioners. We have clarified our choice of this window of time in lines 395-396. We stress, however, that lead week is unrelated to the initial conditions of the model-inference system.

*Whether that is the case or not, it seems false positives are excluded from independent evaluation as well as true negatives. Again, it is unclear why one would exclude these from a valid prediction model and it becomes extremely difficult to evaluate the results.*

We understand your concern that some forecasts predict no onset for seasons and countries where, in reality, no onset occurs (true negatives), and that some forecasts predict an onset for seasons and countries where, in reality, no onset occurs (false positives), and that we do not consider these forecasts in our analyses. However, as our outcome metrics (peak timing, peak intensity, and onset timing) are not defined when no outbreak occurs, a meaningful assessment of the accuracy of these forecasts is not possible, at least for our outcomes of interest. We have now made this reasoning explicit in lines 397-403.

In other words, while we are interested here partly in whether forecasts can predict the week of onset timing in places where an onset does indeed occur, we are not, at the moment, interested in whether forecasts can predict whether or not an onset occurs. However, we have performed a new sensitivity analysis assessing the model’s ability to predict whether or not onsets will occur. We discuss this analysis more in our response to point 18 below, but we note here that false positives at least were incredibly uncommon.

*The conditional probability is very difficult to even define, much less fairly score. The authors should give a very clear explanation of why this is a meaningful prediction test and how they account for this conditioning in the scoring.*

Log scores are calculated for individual forecasts, so the removal of forecasts made for outbreaks with no observed onset prior to analysis has no impact on the scores assigned to the forecasts that are included in the analysis.

*They should also provide clear quantitative information about how many forecasts are discarded for each of these criterion (and any others). If the fraction is large, as it seems to be from qualitative discussion, I do not think it is possible to make meaningful statements about the models’ absolute or relative predictive capabilities. Please convince me otherwise.*

We have created a new supplementary table (Table S2) that shows how many forecast pairs were removed due to either the network or isolated models producing forecasts with no predicted onset, by observed peak and onset lead weeks. For forecasts of peak timing and intensity, the removal of forecasts due to a forecast not predicting an onset was more common at earlier lead weeks, but the majority of pairs are maintained beginning 4 weeks before the peak. For onset timing, forecasts generated prior to the observed onset often predict no onset, and thus the number of forecasts removed here is relatively high. We note that the results of the Friedman test comparing onset timing accuracy were not significant, likely in part due to the much smaller number of forecasts considered.

Because the observed peak and onset lead weeks are not defined when no outbreak occurs, we simply note that 29 of 198 (14.65%) country-season-(sub)type combinations for which we had observations had no observed outbreak (lines 400-401). Thus, the proportion of forecasts that are generated for country-season-(sub)type combinations with no observed onset is relatively low.

To ensure that the removal of forecasts that predicted no onsets did not impact our results, we repeated our analyses using the Friedman test and including all forecasts for countries with an observed onset (i.e., without removing those forecasts that did not predict an onset). Mean log scores by observed lead week are plotted in Figure S5 G-I, and results are discussed in S1 Text under “Results by Observed Lead Week.” Briefly, results for onset timing were unchanged, while a significant difference in forecast accuracy for both peak timing and peak intensity was found. However, the actual difference between the mean log scores for the network and isolated models was very small, as in Fig 3, and the direction of the association (i.e. the tendency to favor the isolated model, whether significantly or not) was unchanged, so this is likely due in large part to the increase in sample size.

*4. Tuning models without cross validation is generally unacceptable, especially for a sample with so few independent events (years). Again, the text is insufficiently clear, but it appears that the model is tuned with dependent data in a variety of ways. The clearest example seems to be in the section on choosing the scaling factors.*

Thank you for pointing this out. We have recalculated scaling factors such that the scaling for a given season is calculated based on attack rates in all other available seasons (described in lines 326-340), and updated all results accordingly.

*I believe that there are also problems with validating forecast for time of peak intensity by lead weak which can only be determined once the peak week is known. This should be carefully discussed.*

Hopefully some of our responses above have already addressed this concern, but we reiterate here that statistical analyses are performed by observed lead week in order to take advantage of the fact that a single forecast for each country-season-(sub)type combination is generated at each observed lead week for both the network and isolated model, allowing us to conduct a paired statistical analysis. We note that mean log scores for peak timing, peak intensity, and onset timing appear to be fairly similar when plotted by observed lead week (Figure S5 D-F) and by predicted lead week (Figure 3), so we feel that the results by observed lead week are broadly representative of what we see by predicted lead week, as well.

*5. This isn’t really a major issue, and it may have to do with this journal. However, it is incredibly annoying to not have figure captions collocated with the figures. If at all possible, duplicate the figure captions and place them near the figures, or if the journal allows, imbed the figures in the text with the captions.*

Figure captions and figures were submitted in the format required by the journal.

*Minor stuff:*

*1. Line 149: What does it mean to “maintain only countries…” Do you mean you only modeled for these countries?*

Exactly. We have rephrased this line to make this clearer (lines 150-152).

*2. Line 156, 57: Specify what distribution you drew from. Uniform? EAKFs would be much happier with a normal distribution when possible.*

Commuters for each route where data were suppressed or unavailable were drawn using Latin Hypercube sampling over all such routes for the 300 ensemble members. Latin Hypercube sampling was chosen as it is highly efficient at exploring possible combinations of values across routes and seasons. The manuscript has been edited to make this explicit (lines 157-159).

*3. Line 175: These are not technically ‘observations’. They are a gridded analysis that gives a lot of weight to climatology.*

Thank you for pointing this out. We have reworded this line (line 177) accordingly.

*4. Line 208: I do not believe you have defined AH yet.*

You are correct. As this isn’t an abbreviation we seem to use again, it has been written out as “absolute humidity” (line 211).

*5. Line 239: It would be clear to also mention how you initialize the state variables at this point.*

We agree and have added the relevant information (lines 246-248). Specifically, we use Latin Hypercube sampling.

*6. Line 240: Again, define the distribution. I assume it is uniform between these bounds. Note again that the EAKF is really designed to work with normal distributions. It is always a challenge to deal with parameter estimation, especially if the quantity is bounded, and you should probably note this or provide a reference.*

With regard to the distribution, see our response to the previous comment.

We agree that our approach to parameter estimation warrants more attention in the manuscript and have added details accordingly (lines 281-284). Specifically, we first note that our priors are informed by the literature. However, the EAKF is capable of exploring parameter values outside of these initial bounds, so estimated parameter values are not strictly constrained by these priors. Aside from the initial state and parameter selection, the only limits we impose on state and parameter values are to ensure that inferred values are neither physically impossible (e.g., a negative number of infected individuals) or otherwise biologically implausible (e.g., D < 0.5 days).

*7. Line 242, also state the variables (like S) from the previous equation here rather than just text names.*

We agree that this makes the section clearer. We have added the variable names (line 251).

*8. Line 243: Define the system parameter set very clearly here and include the variables from the previous equations.*

We have edited the line accordingly (line 252).

*9. Line 251: This is an inaccurate description of what is in X (see major point). State precisely what the vector X includes. A set of state variables for each population subdomain and also parameters? Be mathematically precise.*

Thank you again for pointing this out. As described above, we have more clearly explained *X* in the revised manuscript (lines 263-275).

*10. Line 265: Weeks 44 through 69 did not make sense to me. Clarify.*

Week 44 refers to the 44th week of the year (corresponding to late October), while week 69 refers to the 16th or 17th week of the following year (corresponding to mid April). We have added a similar explanation to the manuscript to make this more clear (lines 303-304). We have also better defined an influenza “season” earlier in the text when we first introduce the influenza data (lines 130-132).

*11. Line 267: You should specify why you chose 300 members. This might fit in with a sentence addressing the absence of localization in your EAKF implementation.*

Thank you for the suggestion. We have added a sentence explaining this (line 293-295).

*12. Line 271: This is related to a major concern. One would expect you to forecast that it has little impact rather than concluding it has little impact and not forecasting it.*

We have attempted to address this above, under major concerns. Our model-inference system is not designed to predict whether or not a specific (sub)type will dominate in several countries, but rather to forecast the timing (both peak and onset) and intensity of outbreaks once there is evidence of its emergence.

*13. Line 280-81. This statement is fundamentally incorrect as your equation (3) points out. Here, I assume that the “data” means observations and that the observations are, of course, not of your specific variables in X. You need to define O=f(X) in this section (see major comment).*

Your assumption here is correct (see our response to the following point). We have attempted to clarify our original statement to say that the EAKF requires that the raw data be transformed in some way, such that both our observations (*O*) and our observed state variables (*X*) represent influenza incidence rates (lines 318-319).

We have also more explicitly defined *O* in this section, per your suggestion (line 333-335).

*14. Line 282: You are not being consistent with the use of the terms ‘observations’ and ‘data’. You have previously defined observations and have used O in equation (3). Here, I believe ‘data’ would be better replaced by ‘observation’. Whatever the case, be clear and consistent.*

Thank you for pointing this out. We have edited the manuscript so that “observations” consistently refers to the scaled observations we use for model inference and forecast assessment (i.e., *O*), while “data” is used to refer to the raw, unscaled data obtained from the WHO. State variables in *X* are referred to as “observed states variables.”

*15. Line 285: This appears to be where cross validation would be appropriate. Otherwise, you will be overfitting by including the year being forecast in the derivation of the calibrating statistics.*

Please see our response to major comment 4 above.

*16. Line 303-04: I have no idea what this sentence is about. What is ‘training’ data. What were these weeks? How does this relate to the EAKF analyses?*

We have edited the wording here to explain that the model was trained through assimilation of 5-30 weeks of observations, and have further rearranged the sentence to make it clearer that this assimilation was done using the EAKF (lines 346-348), as described for the network model earlier in the manuscript.

*17. Line 325-29. This is unclear. Obviously, you can generate forecasts for anything that is a function of X at any time after the initial time. You can only validate forecasts when you have observations. An isolated model can also predict anything at any lead time. Are you making a statement that you don’t generate forecasts from times with no observations at the intial/analysis time? Please clarify.*

Because forecasts at some week *t* are produced by first iteratively assimilating all observations up to and including the observation at week *t*, per Equation 3, then running the SIRS model forward using the most recently inferred states and parameters, it is impossible to generate a forecast at week *t* using the isolated model for a country if no observations are available. We have added an explicit note to this effect (lines 348-351), which we hope clarifies this point.

*18. Line 340-41. This is a very odd statement. You are conditioning your interest on the forecast output. This is statistically unusual; could you even write down a conditional probability expression for this? I also think it’s inherently false. If you are interested in forecasts of onset, then no onset is the negation of that so it provides information.*

We hope that our responses to major comment 3 addresses your concern here. To summarize, we expect our model to be capable of forecasting influenza activity patterns in the presence of influenza signal, but not of reliably predicting whether or not an outbreak will occur in the absence of signal. We therefore focus on forecasts predicting outbreak occurrence, and assess whether these forecasts are capable of predicting key characteristics of ensuing outbreaks.

However, given that all twelve countries will not necessarily experience an outbreak of a given (sub)type, even when that (sub)type is causing widespread activity in other countries during a given season, it is interesting to consider whether the model accurately predicts which countries will and will not experience an outbreak once the outbreak has already begun. For this reason, we conducted a sensitivity analysis which explored, by calendar week, the sensitivity and specificity of binary onset predictions (onset/no onset), starting the week of the first observed onset for a season/(sub)type. Briefly, we find that sensitivity is low at early weeks, but increases with time; specificity is consistently high. In other words, the model was very unlikely to predict an onset when no onset would ultimately be observed, but often predicted no onset for countries where an onset would eventually occur. See “Binary Onset Forecasts” in S1 Text, as well as S13 Fig, for more details.

Future work may benefit from exploring tools and methods that may allow more accurate predictions of when an outbreak of a given (sub)type may or may not occur. However, we disagree that our model’s difficulty in predicting non-events detracts from its ability to predict the timing and intensity of events, given that it predicts that the event will occur (see our response to point 23 below for further elaboration). We know of this limitation of our methods, and therefore focus on accuracy for outbreaks that are actually observed.

*19. Line 344-347. This is unclear. Sounds like you didn’t compare the same forecasts for each country for your multi-country system. That seems unusual.*

With this process, it is possible that different runs are chosen for different countries. However, as we limit our analysis to forecasts predicting an onset, it is often impossible to choose the same forecast run for every country, as certain runs may predict an onset in some countries but not others. By repeating the Friedman test with 1000 random combinations of runs (up from 100 in our initial submission), we attempt to account for any differences in results that may arise based on which runs are chosen, and only consider results to be significant if the median p-value from the 1000 runs falls below 0.05. Thus, while statistical significance depends on which runs are chosen, results are only considered to be significant when different permutations consistently yield significant results favoring one of the models over the other.

*20. Line 377: ‘closely fitting’ This statement is meaningless. Make a quantitative statement or leave it out. Same for line 379 “appear to be well-fit”*

We agree and have supported the former statement using Pearson’s correlation coefficients (lines 449-458). We have removed the sentence about peaks being well-fit, and instead rely on root mean square errors and correlation coefficients to support our assertion that outbreaks are well-fit overall.

*21. Line 385: Only mention of filter divergence (see major comment). You can evaluate quantitatively if the filter is diverging, not only at the end, but also earlier. Clearly needs to be done to have any trust in the system.*

Please see our response to major comment 2 above.

*22. Line 395-96. This was not clear enough. I am not sure if these are forecasts, analyses, free runs.*

We have clarified that each line represents the mean model fit for a single run of the model-inference system, and have explicitly tied this back to Equation 3 (lines 469-476).

*23. Line 408-409. This was perhaps the most troubling sentence about the exclusion of some forecasts. Those that do not predict an onset are ‘unreliable’. If you mean this, that the model cannot predict cases where there is no onset, it is very difficult to envision a dynamical system where a model can do something meaningful about predicting an event, but not its inverse. I’d need to see a discussion of why this could be the case to have any confidence in the results.*

An analogy to hurricane forecasting is apt here.

In this manuscript we do not explore forecasts of incidence (cases 1, 2, 3 weeks in the future), but instead focus on events (onset, peak timing and peak intensity). We do not mean to imply that it cannot predict cases, only that early in the season around the time of onset, the system is not as well constrained as there have been fewer weeks of observations for assimilation, and has a harder time predicting the onset event.

Again, the forecast of onset is made before robust transmission when there is less certain signal for system optimization; consequently, we expect prediction of onset to be a more challenging problem. This type of forecast is less like prediction of a pressure field and more like hurricane forecast. Prediction of easterly waves or whether an easterly wave seeds tropical cyclogenesis is analogous to the prediction of influenza onset, and prediction of the intensification and path of a tropical depression, storm or hurricane is analogous to prediction of peak timing and peak intensity. In both instances, these outcomes are different problems working at different points in the lifecycle of an event (hurricane, disease outbreak). That there is greater accuracy at different points of the lifecycle reflects observational fidelity, understanding of the underlying processes and depiction of those processes. For influenza, we do not have sufficient observation of the sub-country activity and events that support a shift from virus introduction and stuttering chains of transmission to sustained local transmission and more rapidly increasing incidence. The intention here was to determine whether the linkage of countries via commuting and travel would help support forecast of any of the outcome phenomenon, but particularly onset, by using information from neighboring or well-connected countries to better constrain mixing among European countries.

*24. Line 412: This needs to be much more quantitative. A handful of dots covering large ranges is insufficiently informative when you are talking about rejecting many forecasts. I suggest a plot that includes two vertical axes, one plotting the metric and the other plotting the number of retained forecasts.*

Thank you for the suggestion. We have modified Figure 3 in the main text accordingly, although we have elected to plot the log scores, and specify the number of forecasts at each lead week in table form instead by expanding our original Table 1.

We emphasize that differences in the number of forecasts at a given lead week in this figure are not only due to the removal of forecasts that did not predict an onset, but also to the fact that we are considering the predicted lead week, which is partially dependent on the week that each forecast predicts the peak will be.

*25. Line 447. Another troubling sentence. If you are really only retaining a few forecasts, and rejecting most, it is difficult to understand how the predictions can be valid.*

Our phrasing was unclear. You are correct, and in fact what you’ve said is exactly the point we intended to make in this sentence: because so few forecasts are retained, we do not compare onset timing forecasts by country. We have clarified this (lines 521-523).

*26. Line 454-56. Comparing forecast error and spread would be a more traditional and robust statistic than a range. Motivate why you chose the range.*

Our use of the word “range” here was wrong, and we have edited the sentence accordingly (line 535). What we meant to say is that we used several different prediction intervals (25%, 50%, 80%, and so on) in our assessment of calibration.

*27. Line 462: Yet another concern about rejecting forecasts.*

Please see our previous comments. Here, as we are interested in whether forecasts accurately predict the week of outbreak onset, we necessarily discard forecasts that predict no onset week at all.

*28. Line 469: Units (weeks) should be made clear in the figure.*

Thank you for catching this. We have edited the figure accordingly.

*29. Line 472: Grey line was very hard to see.*

We have edited the figure and darkened the line in question.

*Reviewer #2: Kramer, Pei, and Shaman extend the SIRS-EAKF framework to predict the onset timing, peak timing, and peak intensity of several subtypes of influenza in 12 European countries, and evaluate whether incorporating travel patterns between the countries improves forecasts. While their forecasts incorporating travel do not substantially improve forecasts, as seen in the group’s prior efforts for the US and New York City, the authors provide a remarkably thorough discussion of the model assumptions and the shortcomings of the data.*

Thank you.

*All of the major claims comparing the network model (using travel data) and the isolated models are either well-founded or addressed in the discussion. One quibble I might have would be that no baseline model is used as a general comparison for forecast accuracy (e.g. an ARIMA or a model of historical averages). However, the authors avoid making any statements about the overall accuracy of their models and correctly restrict their statements to comparisons between the presented models.*

Thank you for the suggestion. We have added a baseline forecast based on historical expectance to the main text, as described in lines 356-365. Specifically, forecasts for a given country, (sub)type, and season are based on the distribution of observed values of the metric of interest (peak timing, peak intensity, or onset timing) among all other seasons for that same country and (sub)type. We show that both the network and isolated models consistently outperform these historical forecasts (lines 525-530), and discuss the implications of these findings in the context of our work (lines 566-575).

*The authors provide the data (either explicitly in the supplement or by providing the source in the text) and methods required to run the analysis, though I would also appreciate it if they made the corresponding code freely available as well.*

Thank you for pointing this out. We have been working to clean up the code, and it can now be found at: <https://github.com/sarahckramer/euro_flu_network>
